# Understanding socio-labor inclusion among young adults with autism spectrum and mental disorders: preliminary findings

**DOI:** 10.1192/j.eurpsy.2023.939

**Published:** 2023-07-19

**Authors:** M. S. Burrone, M. J. González, M. T. Solís-Soto, P. Valenzuela, L. Rojas, L. Colantonio, C. Cortés, C. Pérez

**Affiliations:** 1 Institute of Health Sciences, Universidad de O´Higgins, Rancagua, Chile; 2 University of Alabama at Birmingham, Birmingham, United States; 3 Institute of Social Sciences, Universidad de O´Higgins, Rancagua, Chile

## Abstract

**Introduction:**

Previous studies suggest that adults with mental disorders (MD) or Autism Spectrum Disorder (ASD) are more likely to be unemployed than those without MD. However, it is unclear whether working adults with MD or ASD perceive the same effort-reward balance as their counterparts without MD or ASD.

**Objectives:**

To analyze labor conditions and to identify factors associated with effort-reward imbalance among young adults with ASD, MD and those from the general population (GP).

**Methods:**

A qualitative and quantitative study design is being conducted to analyze the rates of employment among young adults with ASD, and to identify factors associated with employment rates (Fondecyt ID11201028.). As part of this study, we conducted a quantitative analysis in young adults 16 to 30 years of age in two regions of Chile between August and October, 2022. Young adults with MD and ASD were compared with adults of similar age recruited from the GP. We applied a questionnaire to collect data on participant’s sociodemographic information, autonomy level and employment status. We applied the short Spanish version of the effort–reward imbalance (ERI) and overcommitment (OC) questionnaire, which has been widely used in Latin American countries.

Chi-square test was used and the Kruskal Wallis H Test was applied to compare among groups. The statistical significance was set at P<0.05.

**Results:**

Overall, 422 participants were included in the analysis (mean age 22±3.2, 64.2% women, 65.2% students, and 4.4% unemployed). Of the total respondents, 22% of young adults from GP, 17.8% with MD, and 4.8% with ASD were working at the moment of the survey. Regarding autonomy level, a higher proportion of participants with ASD needed support (36.4%), compared with 9.7% and 0.8% of young adults with MD and GP, respectively. Of the population who reported working (n=125), about 56.0% have a permanent job, and 44% a seasonal or occasional job. The median value for the effort–reward ratio was 0.96 (range 0.4–1.8), with no significant differences between the groups. Of those participants working, 44.3% showed an ERI ratio higher than 1, which was higher in participants with ASD (60%). ERI-esteem was significantly different (P=0.01) among ASD (7.0; range 5-8), MD (6.0; range 2-8) and PG (6.0; range 2-8). In the OC questionnaire, young adults with ASD were more likely to think about work (P=0.01) and having trouble sleeping at night due to work issues (P=0.03) than GP and MD groups.

**Conclusions:**

The ASD group showed higher overcommitment and a considerable proportion of subjects at risk of effort-reward imbalance at work, were more likely to think about work at home, and had trouble sleeping thinking about work. Our preliminary results highlight the importance of considering the working conditions of young adults diagnosed with ASD and the need to provide them with enough support to promote labor inclusion.

**Disclosure of Interest:**

None Declared

